# Supplementary material for: The prevalence of Dupuytren’s disease in patients with diabetes mellitus
Source: Commun Med (Lond). 2023 Jul 13;3:96. doi: 10.1038/s43856-023-00332-7 (PMC10345101; doi:10.1038/s43856-023-00332-7)
Supplement: Supplementary file 1 — Supplementary Information [file 43856_2023_332_MOESM1_ESM.pdf]

**Supplementary materials**

**Supplementary Table 1. DD prevalence cases.** Cumulative cases of DD within DM, T1DM, and T2DM cohorts from 2010-2020. The cases of DD within the DM cohort are largely made up of the cases in the T2DM cohort.

| | **Year** | | | | | | | | | | |
|---|---|---|---|---|---|---|---|---|---|---|---|
| | **2010** | **2011** | **2012** | **2013** | **2014** | **2015** | **2016** | **2017** | **2018** | **2019** | **2020** |
| **DM** | 1,974 | 2,441 | 2,977 | 3,686 | 4,635 | 5,749 | 7,105 | 8,507 | 10,445 | 12,482 | 14,193 |
| **T1DM** | 37 | 46 | 51 | 62 | 74 | 80 | 97 | 124 | 146 | 185 | 203 |
| **T2DM** | 1,683 | 2,084 | 2,559 | 3,181 | 4,036 | 5,032 | 6,235 | 7,481 | 9,211 | 11,027 | 12,564 |
