# Supplementary material for: The prevalence of Dupuytren’s disease in patients with diabetes mellitus
Source: Commun Med (Lond). 2023 Jul 13;3:96. doi: 10.1038/s43856-023-00332-7 (PMC10345101; doi:10.1038/s43856-023-00332-7)
Supplement: Supplementary file 3 — Description of Additional Supplementary Files [file 43856_2023_332_MOESM3_ESM.pdf]

Description of Additional Supplementary Files

File name: Supplementary Data

Description: This MS Excel workbook contains the data used to generate Figures 1-4. The Figure 1 worksheet contains the number of male and female subjects at each age in the cohort of subjects with DD. The Figure 2 worksheet contains the prevalence data for DD in subjects with Dm and the overall cohort. The Figure 3 worksheet contains the prevalence data for DD in the DM, T1DM, and T2DM cohorts by year. The Figure 4 worksheet contains the effect size and confidence intervals for each of the comparisons shown in Figure 4.
