# Supplementary material for: The prevalence of Dupuytren’s disease in patients with diabetes mellitus
Source: Commun Med (Lond). 2023 Jul 13;3:96. doi: 10.1038/s43856-023-00332-7 (PMC10345101; doi:10.1038/s43856-023-00332-7)
Supplement: Supplementary file 4 — Reporting Summary [file 43856_2023_332_MOESM4_ESM.pdf]

nature portfolio

Corresponding author(s): Lawrence C. Hurst
Department of Orthopedics, State University of New York at Stony Brook
HSC T18 Room 092
Stony Brook, NY 11794-8181
USA

Last updated by author(s): Jun 29, 2023

Reporting Summary

Nature Portfolio wishes to improve the reproducibility of the work that we publish. This form provides structure for consistency and transparency in reporting. For further information on Nature Portfolio policies, see our Editorial Policies and the Editorial Policy Checklist.

Statistics

For all statistical analyses, confirm that the following items are present in the figure legend, table legend, main text, or Methods section.

n/a	Confirmed	
☐	☒	The exact sample size (*n*) for each experimental group/condition, given as a discrete number and unit of measurement
☒	☐	A statement on whether measurements were taken from distinct samples or whether the same sample was measured repeatedly
☐	☒	The statistical test(s) used AND whether they are one- or two-sided<br>*Only common tests should be described solely by name; describe more complex techniques in the Methods section.*
☐	☒	A description of all covariates tested
☐	☒	A description of any assumptions or corrections, such as tests of normality and adjustment for multiple comparisons
☐	☒	A full description of the statistical parameters including central tendency (e.g. means) or other basic estimates (e.g. regression coefficient) AND variation (e.g. standard deviation) or associated estimates of uncertainty (e.g. confidence intervals)
☒	☐	For null hypothesis testing, the test statistic (e.g. *F*, *t*, *r*) with confidence intervals, effect sizes, degrees of freedom and *P* value noted<br>*Give P values as exact values whenever suitable.*
☒	☐	For Bayesian analysis, information on the choice of priors and Markov chain Monte Carlo settings
☒	☐	For hierarchical and complex designs, identification of the appropriate level for tests and full reporting of outcomes
☒	☐	Estimates of effect sizes (e.g. Cohen's *d*, Pearson's *r*), indicating how they were calculated

Our web collection on statistics for biologists contains articles on many of the points above.

Software and code

Policy information about availability of computer code

Data collection	The TriNetX Platform was used to collect data used in this study.
Data analysis	Data analyses were conducted om the TriNetX platform, which utilizes a combination of JAVA, R, 21, and Python programing languages in addition to Microsoft Excel (version 16.66.1).

For manuscripts utilizing custom algorithms or software that are central to the research but not yet described in published literature, software must be made available to editors and reviewers. We strongly encourage code deposition in a community repository (e.g. GitHub). See the Nature Portfolio guidelines for submitting code & software for further information.

Data

Policy information about availability of data

All manuscripts must include a data availability statement. This statement should provide the following information, where applicable:
- Accession codes, unique identifiers, or web links for publicly available datasets
- A description of any restrictions on data availability
- For clinical datasets or third party data, please ensure that the statement adheres to our policy

The data that support the findings of this study are available from TriNetX but restrictions apply to the availability of these data, which were used under license for

the current study, and so are not publicly available. Data are however available from the authors upon reasonable request and with permission of TriNetX. The source data used to generate Figures 1-4 are available in the Supplementary Data File.

Human research participants

Policy information about studies involving human research participants and Sex and Gender in Research.

Reporting on sex and gender	Gender was considered in the study design and was determined based on EMR data reported in TriNetX. No disaggregated gender data were collected. Gender-based analyses were performed when comparing demographic characteristics of the four cohorts included in the present study in addition to describing the age and gender distribution of patients with Dupuytren's disease.
Population characteristics	The current study included patients in the TriNetX Research Network as of July 17, 2022. The Dupuytren's disease cohort included patients with a diagnosis code for palmar fascial fibromatosis (ICD-10 code: M72.0). The diabetes mellitus cohort included those with a diagnosis code of either type 1 diabetes mellitus (T1DM) or type 2 diabetes mellitus (T2DM) (ICD-10 codes: E10, E11). Two additional cohorts were created to separate patients with T1DM and patients with T2DM. The T1DM cohort included patients with ICD-10 diagnosis code E10, and the T2DM cohort included patients with ICD-10 diagnosis code Ell.
Recruitment	Patients were not recruited for this study. Aggregate EMR data were used to analyze patients based on ICD-10 diagnosis codes.
Ethics oversight	Because this study used only de-identified patient records and did not involve the collection, use, or transmittal of individually identifiable data, this study was exempted from Institutional Review Board approval.

Note that full information on the approval of the study protocol must also be provided in the manuscript.

Field-specific reporting

Please select the one below that is the best fit for your research. If you are not sure, read the appropriate sections before making your selection.

☒ Life sciences ☐ Behavioural & social sciences ☐ Ecological, evolutionary & environmental sciences

For a reference copy of the document with all sections, see nature.com/documents/nr-reporting-summary-flat.pdf

Life sciences study design

All studies must disclose on these points even when the disclosure is negative.

Sample size	The sample size for each of the four cohorts included all patients in TriNetX with relevant ICD-10 diagnosis codes as mentioned above in "Population characteristics." The purpose of this study was exploratory in nature and not to test a hypothesis; therefore, sample size calculation was not necessary.
Data exclusions	No data were excluded from the analyses.
Replication	Criteria used to create the four cohorts in the present study in addition to the statistical analyses performed are clearly outlined and can be replicated using the TriNetX platform. The current study included EMR data from patients included in the TriNetX Research Network as of July 17, 2022. The TriNetX Research Network is consistently updated with new EMR data, so results of an analysis of the same cohorts may differ slightly due to the inclusion of more recent EMR data.
Randomization	Patients were allocated into groups based on diagnosis codes. Age and gender matching was used when comparing prevalence ratios and prevalence differences between cohorts.
Blinding	Blinding was not relevant to this study as it did not involve determining the effectiveness of an intervention and only used aggregate patient EMR data to explore a connection between Dupuytren's disease and diabetes.

Reporting for specific materials, systems and methods

We require information from authors about some types of materials, experimental systems and methods used in many studies. Here, indicate whether each material, system or method listed is relevant to your study. If you are not sure if a list item applies to your research, read the appropriate section before selecting a response.

Materials & experimental systems

n/a	Involved in the study
☒	☐ Antibodies
☒	☐ Eukaryotic cell lines
☒	☐ Palaeontology and archaeology
☒	☐ Animals and other organisms
☒	☐ Clinical data
☒	☐ Dual use research of concern

Methods

n/a	Involved in the study
☒	☐ ChIP-seq
☒	☐ Flow cytometry
☒	☐ MRI-based neuroimaging
